# Therapeutic Education and Pharmacotherapeutic Follow-Up Protocol, a Useful Tool for the Improvement of Patients at Cardiovascular Risk in Community Pharmacies

**DOI:** 10.3390/jcdd12030080

**Published:** 2025-02-20

**Authors:** Pilar Buenavida Jurado, Mª José De la Matta Martín, Mª José Martín Calero, Rocío De la Puerta

**Affiliations:** 1 Community Pharmacy, 06011 Badajoz, Spain; pilarbuenavidaju@gmail.com; 2 Community Pharmacy, 41006 Sevilla, Spain; mjosedelamatta@redfarma.org (M.J.D.l.M.M.); calero@us.es (M.J.M.C.); 3 School of Pharmacy, University of Seville, 41012 Seville, Spain

**Keywords:** community pharmacy, pharmacist, pharmacotherapeutic follow-up, therapeutic education, cardiovascular risk prevention, professional pharmaceutical services

## Abstract

The aim was to determine the influence of a complex intervention based on pharmacotherapeutic follow-up (PTF) and the application of therapeutic education (TE) protocols on the clinical and educational parameters of patients at cardiovascular risk (CVR) attending community pharmacies (CPs). A prospective, longitudinal, randomized, controlled clinical trial was conducted over 6 months in patients from four Spanish CPs, divided into control (CG) and intervention (IG) groups. CG patients received usual pharmacy care and IG patients received a PTF- and TE-based intervention. The sample consisted of 85 elderly patients. After pharmaceutical follow-up of the IG patients, the following results were observed: significant reductions in cardiovascular risk (CVR) (*p* < 0.005), blood pressure (BP) (*p* < 0.05), and sedentary lifestyle (*p* < 0.001), as well as an improved knowledge of CVR and cardiovascular risk factors (CVRFs) (*p* < 0.001). Target values for BP were achieved in 27.2% of patients and for triglycerides in 12.4% of patients. The PTF of the patients showed that 29.2% did not have the expected response to some treatments, while 25% had untreated pathologies and 10% had adverse reactions. The TE protocols related to the patients’ educational needs, applied individually and in conjunction with the PTF, were able to improve their lifestyle habits, their knowledge of CVR, CVRFs, and pharmacotherapy, and their clinical parameters, and, thus, the level of development of their disease

## 1. Introduction

According to the World Health Organization (WHO), cardiovascular disease (CVD) is the leading cause of mortality worldwide [1], and the identification and control of cardiovascular risk factors (CVRFs), such as hypertension (HT), diabetes mellitus (DM), dyslipidemia, obesity, and smoking, among others, remains the best strategy for prevention. Numerous studies point to the importance of patient involvement for optimal disease control, both in terms of adherence to treatment and the acquisition of healthy habits aimed at controlling/reducing CVRFs [2,3,4].

There are numerous publications regarding interventions on CVR in community pharmacies (CPs), aimed at identifying and/or controlling risk factors as a whole or in isolation [5,6,7], and some studies have shown that in patients over 60 years of age with a medium to low level of education, HT, dyslipidemia, diabetes II, and obesity were the most common CVRFs, with an insufficient level of knowledge about their CVR and CVRFs [8]. There are also many articles describing the health benefits of pharmacotherapeutic follow-up (PTF) in these patients [9,10,11]. There is a precedent for comparing the responses of CVR patients who receive PTF with those who receive health education, demonstrating better outcomes in the former group [12]. However, it is more difficult to find papers describing a complex intervention that includes PTF and the use of specific therapeutic education (TE) protocols in this group of patients.

The main aim of TE is not only to inform, but more importantly to educate patients in the skills, knowledge, and behaviors needed to self-manage their chronic conditions and treatments over time [13]. The approach of different types of interventions related to the patients’ educational and psychoeducational needs and aimed at improving knowledge about their disease can bring health benefits [14]. In addition, when patients undergo PTF, which aims to optimize the effects of pharmacotherapy to minimize potential adverse effects [15], a complex intervention is obtained that aims to help patients understand the impact of the disease on their health, the reason for each of the medications they are taking, and the importance of correctly adhering to dosage guidelines. This protocolized intervention makes it possible to identify the patient’s lifestyle, dietary habits, level of knowledge about their medication, and compliance with dosage guidelines, and to assess their CVRFs and degree of CVR. This work can be carried out in the CP, since frequent contact with the patients each time they come to collect their medication allows the pharmacist to offer and provide this type of service [16].

The purpose of our research was to test the influence of a complex intervention over 6 months based on the PTF and the application of TE protocols on the clinical and educational parameters of patients with CVRFs.

## 2. Materials and Methods

### 2.1. Population Studied, Sample Size and Sampling Method

The study was carried out on regular patients of four Spanish CPs, with two in Seville and two in Badajoz. Patients were selected for randomization in two time slots (morning and afternoon). Those who met the inclusion criteria were invited to participate in the study when they went to the pharmacy to collect their medication. They were given information about the procedure to be followed, and those who agreed to participate had to sign the informed consent form by making an appointment with the research pharmacist.

The processing of patient’s personal data was carried out in accordance with the rules of the General Data Protection Regulation (GDPR) in force in Spain since 2018, adapted to the European Union regulation, namely the GDPR (EU) 2016/679 [17].

Patients over 18 years of age with CVRFs and pharmacological treatment for at least one of them, and/or with a history of cardiovascular diseases (CVD) and/or obesity and/or smoking, without cognitive changes that hindered communication and understanding, were included. Pregnant women and patients who did not agree to participate after receiving the required information were excluded. Allocation to the intervention (IG) and control (CG) groups was randomized by an external person.

The initial sample size was *n* = 100 (25 patients/pharmacy). A difference of *p* < 0.050 or greater in favor of IG in CVR reduction after the follow-up period (6 months) was considered clinically relevant. Based on previous studies of similar characteristics and duration, a standard deviation of the change in population CVR of 0.56 was assumed. Finally, values for significance level (α = 5%) and power (β = 80%) were chosen that are commonly used in randomized controlled trials of the same type [9,18]. Using Altman’s nomogram with these data would require a sample size of ≈40 for each group, giving a total of 80 patients. This size was confirmed by exact calculation using the TrialSize package in RStudio. In anticipation of potential dropouts during follow-up, a total of 100 patients were selected, with 50 patients assigned to each group (CG and IG).

### 2.2. Treatment Groups

*Control group (CG):* Patients in this group received usual care from the participating pharmacies.

(a) An initial interview at time 0 (t0) using a validated questionnaire, previously published by our group, was conducted to obtain information on patient demographics, pathologies, hygiene and health habits, weight control, diet and eating habits, pharmacotherapy, allergies and drug alerts, use of natural medicine, food supplements, vitamins or infusions [8]. The interview was repeated after 6 months (t6). Their perception of the effectiveness of their treatment and their experience with pharmacotherapy at t0 and t6 were also analyzed.
(b) Patient knowledge of CVR and CVRFs was assessed using a validated questionnaire with a series of multiple-choice questions (http://dx.doi.org/10.1016/j.aprim.2016.01.005, accessed on 5 January 2016) [19]. Based on the score obtained, patients were classified into two categories: adequate (>6 points) or inadequate (<6 points) knowledge. The difference in patient knowledge between the initial and final interviews was assessed.
(c) Compliance with pharmacological treatment was assessed at t0 and t6 using the Morisky–Green–Levine test [20].
(d) The following parameters were recorded: systolic blood pressure (SBP) and diastolic blood pressure (DBP) in mmHg, glycosylated hemoglobin (HbA1c), total cholesterol (TC), cholesterol—high-density lipoprotein (c-HDL), cholesterol—low-density lipoprotein (c-LDL), triglycerides (TG) in mg/dL, and body mass index (BMI) values at t0 and t6 for the comparison of results.
(e) The degree of CVR at t0 and t6 was recorded using the SCORE table [21].

*Intervention group (IG):* Patients in this group received a specific intervention from the participating community pharmacies.

(a) A baseline interview at time 0 (t0) was conducted using the same questionnaire as described for CG patients [8] to obtain the necessary information from them. Patients underwent a complex intervention in this group based on the PTF and TE, with bimonthly interviews to evaluate their progress: t2 (2 months after the first interview), t4 (4 months), and t6 (final interview).
(b) In this way, the same items described above for CG patients were assessed every two months: (b–e).

The PTF was carried out according to the methodology proposed in the Guide for Professional Pharmaceutical Care Services in Community Pharmacy [16], in order to determine the need, effectiveness, and safety of the medicines received by the patient, to detect and prevent possible treatment problems and, if necessary, to try to resolve them in collaboration with the prescribing physician (return interview). In addition, a TE protocol previously used by our working group in a specialized CVR unit was applied and adapted to patients assessed in community pharmacies [22]. These were divided into 3 types of intervention: (a) related to the patient’s educational and psychoeducational needs, with 11 categories covering healthy habits, disease management skills, disease knowledge and experience, outcome expectations, cognitive and communication function, and level of knowledge about the medication use process; (b) related to the management of pharmacotherapy, with only one category assessing the patient’s experience with their medication. (C) Related to the use of pharmacotherapy, consisting of the six categories that make up the PTF (two for necessity, two for efficacy, and two for safety), as well as one more category to determine the causes of non-adherence.

The bimonthly interview assessed the percentage of patients who started or maintained healthy habits, the percentage who increased their knowledge of CVR and CVRFs, and those who improved their adherence to treatment. In some cases, an additional intervention was needed, and follow-up interviews were also conducted with patients whose doctor made a change after the pharmaceutical intervention.

Pharmacy consultation: Patients selected and randomized to the IG and CG groups were scheduled for an initial 30 min interview in the personalized care area of the pharmacy during pharmacy opening hours. Subsequent bimonthly interviews lasted about 15 min. The contents of the questionnaires were recorded in Microsoft^®^ Excel^®^ 2013 software for prior database creation and subsequent statistical processing. To avoid personal bias, the interviewing pharmacist was the same in all pharmacies.

### 2.3. Evaluation of the Intervention

A randomized, longitudinal, prospective, unblinded, controlled clinical trial was conducted. The duration of the work was from January 2018 to July 2021 (due to the COVID-19 pandemic, the procedure was extended by 6 months).

The variables related to the objective of the project were determined in the CG at the start time (t0) and at the end time (t6). In the IG, these parameters were determined at each of the bimonthly appointments:

- Mean values of decrease in SBP and DBP values obtained according to the protocol proposed by the Spanish Society of Cardiology [23] and using a calibrated and validated Omron M3 Confort (HEM-7134-E, Omron Healthcare, Hoofddorp, The Netherlands) digital blood pressure monitor ( with cuffs adaptable to the arm circumference of each patient.
- Mean values of decrease in lipid profile values of TC, c-LDL, and TG. Roche Laboratories COBAS b101^®^ devices (Roche Diagnostics GmBH, D-68305 Mannheim, Germany) were used, Accu-Chek Safe-T-Pro Plus^®^ lancets were used to perform the punctures, and medical supplies (gauze, 96° alcohol and cotton wool) were provided by each pharmacy.
- Average reduction in HbA1c levels using COBAS b101^®^ devices (Roche Diagnostics GmBH, D-68305 Mannheim, Germany) and the above materials.
- Average BMI reduction and patients reaching normal weight [24]. Patients’ weight and height were measured using mechanical scales in each pharmacy, calibrated and validated with an integrated altimeter.
- Percentage reduction in sedentary patients according to WHO criteria [25].
- Percentage reduction in the number of patients who smoke, by assessing the number of patients who managed to reduce their consumption of cigarettes/day.
- Percentage of patients achieving clinical control targets for better CVR prevention of the parameters SBP, DBP, TC, c-LDL, c-HDL, TG, and HbA1c, defined for each patient according to their clinical condition.
- Level of knowledge achieved by the patient about CVR and CVRFs between t0 and t6.
- Average level of medication adherence: percentage of patients who improved their adherence at the end of the study (t6).
- The pharmacotherapy profiles of the patients were assessed.
- The type and number of educational interventions required for each patient were assessed only in the IG.

At the end of this intervention period, a CG–IG comparison was performed to evaluate the difference in the results obtained between t0 and t6 in the patients of each group.

### 2.4. Statistical Analysis

The free and open-source software RStudio, version 1.1.414, © 2025–2018 RStudio Inc., was used. Within this integrated development environment (IDE), after the usual work of debugging and resolving inconsistencies in the data, most of the calculations and tables were obtained using the compareGroups version 4.0 package. Data archived in Excel were exported to the RStudio software (version 4.0.0.). All results were expressed as means and standard deviations for continuous variables and frequencies and percentages for categorical variables. Continuous variables were analyzed using Student’s *t*-test for two samples, while categorical data were analyzed using Pearson’s chi-squared test. A *p*-value < 0.05 was considered statistically significant.

## 3. Results

### 3.1. Sociodemographic Characteristics of the Sample

Of the initial 100 patients, 15 dropped out of the study for various reasons, such as change of address, lack of interest, or illness of a close relative. The sample was reduced to 85 patients (41 in CG and 44 in IG) distributed among the 4 pharmacies.

The gender distribution in both groups, the level of education, and the type of living arrangements, as well as the mean age of the sample with the age distribution range, are shown in Table 1. Although the sample was initially evenly split between men and women, after the losses, women predominated in the CG (56.1%) and men predominated in the IG (59.1%).

### 3.2. Prevalence of CVRFs and CVR Value

Table 2 shows the percentages and baseline values (t0) of the different CVRFs, as well as the level of prevention (primary and secondary prevention) and the CVR value of the CG and IG. Secondary prevention patients are not included in the CVR calculation as the SCORE table considers them to be at high risk, as they have already suffered a cardiovascular event.

The remaining CVRFs of the two groups at baseline (t0), weight level, BMI, level of physical activity, and tobacco consumption are shown in Table 3.

### 3.3. Level of Individual Patient Knowledge of CVR and CVRFs

Using the test to assess patients’ knowledge of their CVR and CVRFs [19], the mean of the total number of adequately answered questions was 4.3 ± 1.46, corresponding to an inadequate level of knowledge.

### 3.4. Adherence to Pharmacotherapy

The overall mean of patients’ adherence to prescribed medication was 72.4% according to the Morisky–Green test [20].

### 3.5. Prevalence of Pharmaceutical Interventions Related to Educational and Psychoeducational Needs and the Patient’s Pharmacotherapy

The list of all the interventions carried out following the application of the protocols described in Section 2.2. of “treatment groups” in the material and methods is presented in Appendix A (Table A1, Table A2 and Table A3) at the end of the article. The first column indicates the category evaluated, the second column lists each of the items corresponding to the interventions related to the problems identified in the patients of this category, and the third column shows the number and percentage of interventions carried out.

From the results shown in these tables, it was confirmed that 100% of the intervention patients had to be trained in the CVR knowledge category (Table A1). It was also necessary to explain the importance of “Physical activity” to improve the patients’ clinical condition (50.0%), to use motivational interviewing to help the patient feel able to initiate and maintain this healthy habit in the long term (52.1%), and to explain the frequency, duration, and intensity required to achieve the desired results in 60.4% of patients (Table A1). As for the category of “heart-healthy diet”, 66.7% of patients needed to be given an idea of the impact of certain foods and/or ingredients (salt, sugar, fats, alcoholic drinks…) on their pathology, and motivational interviewing was necessary for 60.40%. In addition, 68.8% were offered a list of heart-healthy foods (Table A1).

In the “knowledge and experience about their pathologies” category (Table A1), the most appropriate learning method had to be chosen for each individual (56.2%) in order to teach them about their condition: identification, causes, duration, consequences, and appropriate control. Here, 41.7% needed to be informed and trained about what self-management is and why it is necessary on a regular and long-term basis, and 43.8% needed information about the purpose of self-management in order to be able to assess whether the medicines they were using were effective. It is curious that 12.5% of patients did not want to take the medication because they considered it to be a sign of aging, while 6.25% of patients were afraid to start using medication (Table A2). In Table A3, it can be observed that 25% had an untreated medical condition, while in 29.2% of patients, the medication was not producing an adequate response. In the “medication adherence” category (Table A3), it should also be noted that 27.1% needed education about the importance of adherence. In addition, a careful study of these three tables gives us much useful information about the patients.

### 3.6. Evaluation of the Pharmaceutical Intervention

Table 4 shows the evolution of the two groups, CG and IG, after 6 months of pharmaceutical follow-up. In the IG patients, there was a significant reduction in CVR (*p* < 0.005), BP (*p* < 0.05) and sedentary lifestyle values (*p* < 0.001). Knowledge of CVR and CVRFs also increased in the IG for all patients (*p* < 0.001).

The percentage of patients achieving the target values for each of the CVRFs assessed is shown in Table 5. The control values established by the European guidelines have been taken into account according to the clinical condition of each patient, depending on whether they were diabetic or not and the level of prevention they were in. It is noteworthy that in the IG, the control of SBP levels, which was 52.3% at t0, increased to 79.5% after the intervention, reaching a difference of 27.2%. The percentage of patients with controlled triglycerides also increased by 12.4%.

The pharmaceutical follow-up of the IG patients showed that 29.2% of the patients did not achieve the expected response (ineffectiveness), 25% had untreated pathologies (they needed treatment), and 10% had an adverse reaction. 

## 4. Discussion

The community pharmacist can play an important role in the primary and secondary prevention of CVD and in the detection and control of specific CVD through patient education and counselling programmes that increase patients’ knowledge about the management and safety of their treatments, reviewing their medication, and monitoring clinical parameters [17]. Although systematic reviews of randomized controlled and observational studies have documented improved control of some conditions, such as HT, and a reduction in patient hospitalizations [26,27], more research is needed to quantitatively and qualitatively assess the impact of pharmaceutical interventions on CVD.

A recent study showed that the majority of patients with CVR presenting to the CP are over 60 years of age and have a medium/low level of education, while a high percentage have developed hypertension, are overweight or obese, tend to be sedentary, and have inadequate knowledge of their CVRFs and CVR level [8].

These results led us to apply a complex intervention based on the use of a TE protocol accompanied by PTF, which has previously been applied by our research group and shown to be effective in patients at the Specialized CVR Service of a local hospital in Seville [28].

The results show that 100% of IG patients needed to be given basic knowledge about CVR and CVRFs. A high percentage who was unaware of the benefits of a heart-healthy diet and moderate physical activity were informed of the benefits of a proper diet, provided with graphic and written material to help them improve their habits, and informed and helped to start moderate physical activity, while smokers were informed of the need to reduce and/or stop using tobacco.

Specific goals were set with each patient, and behavioral changes to achieve these goals were encouraged at each session. More than half required motivational interviewing, which is recognized by the American Heart Association as an effective intervention for promoting health-related outcomes [29] and aims to stimulate and convince the patient of the importance of their direct involvement in achieving the desired goals and maintaining them over time.

Problems related to patients’ treatments were also addressed in each interview to assess their effectiveness and to identify and/or prevent adverse effects [16]. Because pharmacists work directly with patients to assess their medication, they are more aware of the need to adhere to treatment guidelines and contribute to optimising the management of their pharmacotherapy [30]. Protocol-based monitoring is particularly beneficial in multi-morbid and polymedicated chronic patients, who are at greater risk of inadequate pharmacological treatment due to continuous transitions in care [31]. In many cases, physician intervention is required to resolve or prevent these inadequacies, creating a highly beneficial pharmacist–patient–physician relationship. Several studies have shown that the involvement of the pharmacist in the healthcare team responsible for the patient’s care leads to improved medication efficacy and safety [32,33,34,35].

This work has shown that the combined use of specific protocols of TE and PTF in IG patients led to a significant reduction in some parameters, such as SBP (*p* < 0.05), CVR (*p* < 0.05), and sedentary lifestyle (*p* < 0.001), as well as a significant improvement in knowledge of CVR and CVRFs (*p* < 0.001) after 6 months of follow-up compared to the CG. In general, a higher number of IG patients achieved control values after pharmaceutical intervention for DBP, TG, HbA1c, and BMI. These results, analyzed in the overall sample and in each of the pharmacies where the study was conducted, show that the work of the community pharmacist can be highly relevant in helping patients to control CVRFs.

The EMDADER HTA study, carried out in Spanish pharmacies, demonstrated the beneficial results of PTF in outpatients with hypertension, with a significant increase of 19.6% in the percentage of patients achieving SBP control targets [9]. In our study, with the combined intervention of TE and PTF, this percentage increases to 27.2%, which could demonstrate the greater efficacy of the procedures used.

PTF combined with TE resulted in an overall significant improvement in the health status of IG patients. This suggests that there is an obvious need to establish broader and more effective educational interventions that are sensitive to the perceptions, attitudes, and skills of individual patients, and that the community pharmacist can play a relevant role in the primary and secondary prevention of CVD.

The number of patients was somewhat limited due to the difficulty of recruiting cv-risk patients in the community pharmacies. The reliability of the information provided by the patients could be questioned if they did not understand the questions well or did not answer them truthfully. Also, the fact that they are regular users of pharmacies could influence the results. This was minimized by the fact that the interviewer was a person outside the pharmacy staff and the same for the whole study.

## 5. Conclusions

The TE protocols related to patients’ educational and psychoeducational needs, applied individually and in combination with the PTF, helped patients to improve their lifestyle habits, CVR and CVRF knowledge, adherence to treatment, and optimization of pharmacotherapy. As such, we must highlight the role of the pharmacist in motivating patients to adopt healthy lifestyle habits in order to improve their state of health.

## Figures and Tables

**Table 1:** Sociodemographic characteristics of the sample. Control and intervention groups (CG, IG).

		CG (*n*:41)	IG (*n*:44)
		*n* (%)	*n* (%)
**Gender**	Male	18 (43.9%)	26 (59.1%)
	Female	23 (56.1%)	18 (40.9%)
**Education**	Primary school	14 (34.1%)	14 (31.8%)
	High school	12 (29.3%)	11 (25.0%)
	Without studies	12 (29.3%)	8 (18.2%)
	University studies	3 (7.32%)	11 (25.0%)
**Living at home**	In company	38 (92.7%)	40 (90.9%)
	Alone	3 (7.32%)	5 (9.09%)
**Age (mean ± SD)**		61.1 ± 10.1	61.6 ± 10.3
**Age distribution range**			
	20–39	0	0
	40–59	15	16
	60–74	23	24
	>75	3	4

SD: standard deviation.

**Table 2:** Prevalence of CVRFs (%): hypertension, diabetes mellitus, and dyslipidemia. Percentage of prevention groups (%). Mean baseline values (t0) of blood pressure (BP), biochemical parameters (TC, c-HDL, c-LDL, TG, and HbA1c), and cardiovascular risk (CVR) (mean ± SD). Control and intervention groups (CG, IG).

	CG (*n* = 41)	IG (*n* = 44)	Significance (*p*-Value)
**Hypertension**	33 (80.5%)	39 (88.6%)	0.458
**SBP**	133 ± 19.6	139 ± 19.6	0.162
**DBP**	78.2 ± 9.69	81.6 ± 11.7	0.153
**Diabetes mellitus**	19 (46.3%)	13 (29.5%)	0.170
**HbA1c^1^**	6.76 ± 1.05	6.75 ± 0.79	0.959
**HbA1c^2^**	5.65 ± 0.49	5.67 ± 0.51	0.894
**Dyslipidemia**	30 (73.2%)	26 (59.1%)	0.255
**TC**	167 ± 39.2	169 ± 40.1	0.808
**c-HDL**	49.4 ± 15.7	51.3 ± 14.7	0.552
**c-LDL**	85.5 ± 30.7	84.1 ± 31.2	0.834
**TG**	173 ± 67.0	170 ± 88.7	0.852
**Prevention**			
**Primary**	29 (70.7%)	32 (72.7%)	1.000
**Secondary**	12 (29.3%)	12 (27.3%)	1.000
**CVR**	1.69 ± 1.07	2.56 ± 2.18	0.050

CVRFs: cardiovascular risk factors; CVR: cardiovascular risk; SD: standard deviation; SBP: systolic blood pressure; DBP: diastolic blood pressure; TC: total cholesterol; c-HDL: high-density cholesterol; c-LDL: low-density cholesterol; TG: triglycerides; HbA1c^1^: glycosylated hemoglobin in patients diagnosed with diabetes mellitus; HbA1c^2^: glycosylated hemoglobin in undiagnosed patients with diabetes mellitus.

**Table 3:** Prevalence of other CVRFs (%): grade of weight/obesity, sedentary lifestyle, and smoking habit. BMI values (mean ± SD). Control and intervention groups (CG, IG).

	CG (*n* = 41)	IG (*n* = 44)	Significance
	*n* (%)	*n* (%)	*p*-Value
**Normal weight**	2 (4.88%)	4 (9.09%)	0.859
**Overweight grade I**	6 (14.6%)	8 (18.2%)	
**Overweight grade II**	11 (26.8%)	8 (18.2%)	
**Type I obesity**	11 (26.8%)	13 (29.5%)	
**Type II obesity**	6 (14.6%)	7 (15.9%)	
**Type III obesity**	5 (12.2%)	3 (6.82%)	
**Sedentariness**	21 (52.5%)	24 (54.5%)	1.000
**Ex-smoker**	17 (41.5%)	19 (43.2%)	0.878
**Smoker**	8 (19.5%)	10 (22.7%)	
**Non-smoker**	16 (39.0%)	15 (34.1%)	
**BMI (mean ± SD)**	32.3 ± 6.03	31.0 ± 5.19	0.283

SD: standard deviation; CVRFs: cardiovascular risk factors; BMI: body mass index.

**Table 4:** Evolution of inter- and intra-group (CG and IG) comparison at baseline (t0) and final (t6) time during follow-up of biochemical parameters (CT, HDL-C, LDL-C, TG, and HbA1c), body mass index (BMI), and cardiovascular risk (CVR), as well as the degree of knowledge about CVR and CVRFs (mean ± SD). Evolution of the prevalence of sedentary patients (%).

	t_0_		t_6_		Difference			
	CG	IG	CG	IG	CG	IG	*p*-Value	*n*
**SBP**	133 ± 19.6	139 ± 19.6	130 ± 16.8	130 ± 15.5	−2.32 ± 12.4	−8.84 ±16.0	0.038	85
**DBP**	78.2 ± 9.69	81.6 ± 11.7	78.2 ± 9.83	80.9 ± 21.3	0.02 ± 7.52	−0.68 ± 18.7	0.818	85
**TC**	167 ± 39.2	169 ± 40.1	181 ± 37.5	171 ± 34.6	12.1 ± 30.7	2.09 ± 24.7	0.109	83
**c-HDL**	49.4 ± 15.7	51.3 ± 14.7	51.6 ± 14.1	51.5 ± 14.2	3.54 ± 11.1	1.30 ± 9.11	0.314	85
**c-LDL**	85.5 ± 30.7	84.1 ± 31.2	93.9 ± 34.4	87.6 ± 30.0	7.12 ± 24.2	2.43 ± 22.4	0.366	82
**TG**	173 ± 67.0	170 ± 88.7	177 ± 70.5	159 ± 87.2	2.20 ± 58.9	−10.30 ± 103	0.497	83
**HbA1c^1^**	6.76 ± 1.05	6.75 ± 0.79	7.03 ± 0.98	6.65 ± 0.96	0.27 ± 0.76	−0.10 ± 0.84	0.222	31
**HbA1c^2^**	5.65 ± 0.49	5.67 ± 0.51	5.64 ± 0.46	5.65 ± 0.96	−0.03 ± 0.31	0.00 ± 0.28	0.777	52
**BMI**	32.3 ± 6.03	31.0 ± 5.19	32.1 ± 6.30	30.8 ± 5.25	−0.21 ± 0.89	−0.22 ± 1.01	0.980	85
**CVR**	1.69 ± 1.07	2.56 ± 2.18	1.69 ± 1.00	1.91 ± 1.42	−0.07 ± 0.46	−0.78 ± 1.16	0.003	61 *
**Degree of knowledge**	4.39 ± 1.45	3.98 ± 1.44	4.66 ± 1.32	8.00 ± 0.00	0.27 ± 0.95	4.02 ± 1.44	<0.001	85
**Sedentariness (%)**	21 (52.5%)	24 (54.5%)	26(65.0%)	7 (15.9%)			<0.001	84

* Patients in secondary prevention, already considered as being at high risk according to SCORE, have not been included in the calculation of CVR.

**Table 5:** Percentage of patients who, according to their clinical condition, reach the target figures for the control of each of the parameters related to CVRF (SBP, DBP, HDL-C, LDL-C, TG, HbA1c, and BMI). Control and intervention groups (CG, IG).

	CG	CG	Difference	IG	IG	Difference
	*n* = 41	*n* = 41	Δ (t_6_ − t_0_)	*n* = 44	*n* = 44	Δ (t_6_ − t_0_)
	t_0_	t_6_		t_0_	t_6_	
**SBP**	26 (63.4%)	30 (73.2%)	9.8%	23 (52.3%)	35 (79.5%)	27.2%
**DBP**	36 (87.8%)	36 (87.8%)	0.0%	34 (77.3%)	37 (84.1%)	6.8%
**c-HDL**	27 (67.5%)	31 (75.6%)	8.1%	33 (76.7%)	35 (79.5%)	2.8%
**c-LDL**	21 (52.5%)	15 (36.6%)	−15.9%	21 (48.8%)	20 (45.5%)	−3.3%
**TG**	16 (40.0%)	15 (36.6%)	−3.4%	22 (51.2%)	28 (63.6%)	12.4%
**HbA1c**	19 (47.5%)	21 (51.2%)	3.7%	28 (65.1%)	29 (65.9%)	0.8%
**BMI**	2 (4.88%)	3 (7.32%)	1.77%	4 (9.09%)	5 (11.4%)	2.3%

CVRFs: cardiovascular risk factors; SBP: systolic blood pressure; DBP: diastolic blood pressure; c-HDL: high-density cholesterol; c-LDL: low-density cholesterol; TG: triglycerides; HbA1c: glycosylated hemoglobin; BMI: body mass index.

## Data Availability

Data supporting the reported results are available from the authors on request.

## Appendix A

**Table A1:** Prevalence of pharmaceutical interventions related to educational and psychoeducational needs.

Category Evaluated (Problem Detected)	Pharmaceutical Interventions	Frequency of Intervention *n* (%)
**Physical activity (PA)**		
Patients do not know which PA to do	Explain and help to choose an appropriate PA	21 (43.8%)
Patients do not understand the importance of doing PA	Explain the importance of PA to improve clinical condition	24 (50.0%)
Patients are not motivated to engage in PA	Motivational interviewing to help start and maintain PA	25 (52.1%)
Patients have limitations in carrying out PA (physical, time, fear)	Develop a specific plan according to their limitations	11 (22.9%)
Patients perform PA, albeit inappropriately	Indicate frequency, duration, and intensity for desired results	29 (60.4%)
	Teach patients how to balance diet and medication during PA	2 (4.17%)
	Inform patients about safety precautions for performing AP	6 (12.5%)
**Heart-healthy diet (HHD)**		
Patients are unaware of the appropriate diet for their clinical condition	Explain the effect of salt, sugar, alcoholic beverages, etc., on their pathologies	32 (66.7%)
Patients do not understand the importance of an HHD to their health condition	Motivational interviewing for the patient to initiate and maintain an HHD	29 (60.4%)
Patients are not motivated to have an HHD	Development of a meal plan according to the patient’s preferences and financial conditions	11 (22.9%)
Patients have cultural, emotional, or financial barriers	Offer a list of heart-healthy foods	33 (68.8%)
**Smoking**		
Patients do not know the consequences of smoking on their pathologies	Explain the consequences of smoking and contrast the perceived benefits	12 (25.0%)
Patients think smoking 5 to 10 cigarettes is safe	Present possibilities for help: psychological support and pharmacotherapy	6 (12.5%)
Patients do not know what to do to quit smoking	Teach skills needed to quit smoking depending on the phase where they are	7 (14.6%)
Patients are unaware of the existence of treatments to quit smoking	Minimum intervention (3 min) at the end of the initial interview (provide written support material)	7 (14.6%)
Phase of abandonment in which the patient finds himself	Insist on reducing consumption per day. Motivate towards change. Deliver support material	2 (4.17%)
Smoking level (number of cigarettes per day)	Refer the patient to the specialized smoking unit for pharmacotherapy	5 (10.4%)
	Help improve adherence to treatment, insist on total abstinence, help recognize risk situations	To all smokers
**Skills and competencies to manage the disease**		
Patients do not know how to self-control and record their parameters	Demonstration on biochemical parameter measurement techniques	25 (52.1%)
Patients do not know how to evaluate risk situations and decide what to do	Request that patients perform the techniques for measuring the parameters of their diseases	26 (54.2%)
Patients do not know how to use medications because of the complexity of the dosage form	Teach them how to record the measured parameters	26 (54.2%)
	Explain the importance of the measures and the appropriate schedules	24 (50.0%)
	Teach what to do in risky situations	24 (50.0%)
	Ask the patient to teach how to use medications at home and make necessary corrections	1 (2.08%)
	Teach patients how to use medicines in different dosage forms	To all interviews
**Knowledge about CVR**		
Patients do not know what cardiovascular risk is	Teach patients what CVR and CVRF are	48 (100%)
Patients do not know the real cardiovascular risk	Teach patients how to estimate the risk and how important it is to know the actual CVR	48 (100%)
Patients are unaware of the different CVRFs they suffer from and their control objectives	Teach patients which CVRFs present and their control objectives	48 (100%)
	Teach patients the importance of controlling each CVRF	48 (100%)
**Knowledge and experience about their pathologies**		
Lack of basic knowledge about the pathologies that patients present	Teach about pathologies: causes, duration, consequences, and adequate control	27 (56.2%)
Patients do not know what self-control is for	Teach what self-control is and the importance of doing it periodically and in the long term	20 (41.7%)
Patients do not perform self-control because thinks it is not necessary	Relate the goal of self-monitoring to the effectiveness of the medications they have	21 (43.8%)
Patients describe negative experiences of their own or of other people with these pathologies	Discuss the causes of negative experiences of the disease	10 (20.8%)
Patients make mistaken assessment of their physiological state	Teach them how to assess their physiological state and what to do in case of imbalance	4 (8.33%)
**Outcome expectations**		
Patients do not know the natural history of their disease	Teach the natural history of the disease and when it is or is not controlled	4 (8.33%)
Patients feel false expectations in the results with the use of medication or alternative therapies	Explain the expected short-, medium-, and long-term outcomes with appropriate use of pharmacotherapy, lifestyle changes, and non-pharmacological measures	5 (10.4%)
Patients do not know what to expect now that they have a chronic illness	Explain the therapeutic goals they need to achieve for each drug and in how long	12 (25.0%)
	Foster and strengthen trust in the treatment and in the doctor	4 (8.33%)
**Self-efficacy**		
Patients have low self-efficacy	Teach them the results of their learning during follow-up: illness, medications, self-control	7 (14.6%)
Patients do not know how to manage their disease	Help them perceive that they are self-effective in controlling their disease	5 (10.4%)
Patients have a lack of perception about its effectiveness	Teach and help them perceive that they are self-effective in controlling their disease	1 (2.08%)
**Cognitive and Communication Function**		
Patients do not prepare for the consultation and forget to discuss important things about their health condition	Teach the patient to prepare before going to appointments, writing reminders of what is important	2 (4.17%)
Patients forget the guidance of the health professional at the end of the consultation	Teach them how to request a report at the end of the consultation and not be left with doubts	2 (4.17%)
Patients do not know how to recognize and express their needs	Encourage the patient to call the professional when in doubt	3 (6.25%)
	Teach them to speak assertively with the professional about doubts and needs	3 (6.25%)
	Help the patient improve their knowledge of diseases, treatments, and self-efficacy	1 (2.08%)
**Risk perception**		
Patients do not realize the seriousness of their clinical condition	Work with patients to make them aware of their actual health condition and the risks to which they are exposed	9 (18.8%)
**Knowledge about the process of using medications**		
Patients do not know what disease each of the drugs is used for	Explain to the patient about the medication: its name, what disease it is used for, dosage, how to take it and until when, and common adverse effects	8 (16.7%)
Patients do not know the names of the medications they take	Teach them how to use medicines correctly: demonstration, written information	2 (4.17%)
Patient do not know the correct use process	Teach the importance of using all medications correctly by relating them to their effects on disease control	5 (10.4%)

**Table A2:** Prevalence of pharmaceutical interventions related to patient-centred pharmacotherapy management.

Category Evaluated (Problem Detected)	Pharmaceutical Interventions	Frequency of Intervention *n* (%)
Experience with Pharmacotherapy		
Patients do not know the usefulness of the prescribed medication	Listen to and acknowledge the different feelings expressed by patients	3 (6.25%)
Patients are afraid to start using a drug	Pay attention to and try to assess the expectations and concerns of patients	3 (6.25%)
Patients doubt the need to use one medication or add another to their treatment	To make patients understand the need of medication	3 (6.25%)
Patients believe that taking more medication means that their health is getting worse	Undoing the patient’s mistaken beliefs about their health status and treatment	1 (2.08%)
Patients have incorrect expectations about the use of their medication	Help patients to understand and accept that they have a chronic disease which can be managed with the necessary pharmacotherapy, with benefits and adverse reactions	2 (4.17%)
Patients fear of having their medication withdrawn or their drug therapy changed	Teach patients when the medication is not being effective and needs to be changed	2 (4.17%)
Patients do not want to increase the dose for fear of adverse effects	Perform negotiation methods with the patient to make changes in pharmacotherapy	3 (6.25%)
Patients do want to live with the negative effects of their medication	Offer and help the patient choose alternatives that best meet their expectations and precautions	2 (4.17%)
Patients fear of using several medicines for the same disease	Share with patients about more effective and/or safer medication alternatives	1 (2.08%)
Patients do not want to withdraw or decrease the dose for fear of losing its beneficial effects	Respect the patient’s autonomy in making their own decisions	1 (2.08%)
Because of previous negative experiences, patients do not believe they should take the medication	Condescending to the decisions made by the patient	1 (2.08%)
Patients do not use medication as prescribed or make dose adjustments without valid criteria	Motivate them to make lifestyle changes to increase the effectiveness of medications	2 (4.17%)
Patients do not want to take medication because they still do not accept the illness	Teach them about their illnesses and about the use of their medication	3 (6.25%)
Patients do not want to take the medication because it is a sign of aging	Teach them the benefits of pharmacotherapy in managing their disease and preventing future complications	6 (12.5%)
Patients do not like to take medication	Increase patient self-efficacy in disease control and pharmacotherapy	1 (2.08%)

**Table A3:** Prevalence of pharmaceutical interventions related to drug-centred pharmacotherapy management.

Category Evaluated (Problem Detected)	Pharmaceutical Interventions	Frequency of Intervention *n* (%)
**Unnecessary medication**		
There is no valid clinical indication for pharmacotherapy at this time	Inform the doctor about the need to stop the unnecessary medication	2 (4.17%)
The pharmacotherapy used is to treat an avoidable adverse effect	Inform the doctor about the need to stop the unnecessary medication and correct the adverse effect	1 (2.08%)
**Need for pharmacotherapy**		
Patients have an untreated medical condition	Inform the doctor of the need to start the necessary treatment	12 (25.0%)
The disease needs to add a drug to achieve synergistic effect	Inform the doctor of the need to add synergistic medication	4 (8.33%)
There is a need for preventive pharmacotherapy	Inform the doctor of the need for added preventive pharmacotherapy	7 (14.6%)
**Ineffective medication**		
The medication does not produce the desired response	Inform the doctor of the need to withdraw the medication and start a new treatment	14 (29.2%)
**Adverse reaction**		
The medication is producing an adverse reaction	Inform the doctor of the need to discontinue the medication responsible for the adverse reaction or allergy	1 (2.08%)
Safer medication is needed because of existing risk factors	Inform the doctor about the need to stop and switch to another, safer medication	3 (6.25%)
The medication is contraindicated due to existing risk factors	Inform the doctor about the need to stop and switch to another, safer medication	1 (2.08%)
**Medication adherence**		
Patients prefer not to use medication	Assess whether the causes are related to the patient’s subjective experience with their medications	11 (22.9%)
Patients do not use medicine because they do not understand the instructions for use	Talk to patients about adherence and its benefits	13 (27.1%)
Low adherence to pharmacotherapy due to forgetfulness	Simplifying the therapeutic regimen when possible	1 (2.08%)
Patients are adherent only to certain medications	Teach patients to understand the use of all their medication	2 (4.17%)
Patients do not have access to their medications	Associate taking their medications with a regular daily activity	9 (18.8%)
	Indicate to patients reminders of the times of taking the medications	3 (6.25%)
	Talk to patients about previous adverse reactions and explain how to prevent them from recurring	1 (2.08%)
	Negotiate goals for adherence to each medication and promote positive reinforcement	2 (4.17%)
